# Application of decellularized bone matrix as a bioscaffold in bone tissue engineering

**DOI:** 10.1186/s13036-021-00282-5

**Published:** 2022-01-05

**Authors:** Halimeh Amirazad, Mehdi Dadashpour, Nosratollah Zarghami

**Affiliations:** 1 Department of Medical Biotechnology, Faculty of Advanced Medical Science, Tabriz University of Medical Sciences, Tabriz, Iran; 2 Department of Biotechnology, Faculty of Medicine, Semnan University of Medical Sciences, Semnan, Iran; 3 Biotechnology Research Center, Semnan University of Medical Sciences, Semnan, Iran; 4 Deparment of Medical Biochemistry, Faculty of Medicine, Istanbul Aydin Universioty, Istanbul, Turkey; 5 Department of Clinical Biochemistry and Laboratory Medicine, Faculty of Medicine, Tabriz University of Medical Sciences, Tabriz, Iran

**Keywords:** Decellularized extracellular matrix, Bioscaffold, Tissue engineering, Bone regeneration

## Abstract

Autologous bone grafts are commonly used as the gold standard to repair and regenerate diseased bones. However, they are strongly associated with postoperative complications, especially at the donor site, and increased surgical costs. In an effort to overcome these limitations, tissue engineering (TE) has been proposed as an alternative to promote bone repair. The successful outcome of tissue engineering depends on the microstructure and composition of the materials used as scaffold. Decellularized bone matrix*-*based biomaterials have been applied as bioscaffolds in bone tissue engineering. These biomaterials play an important role in providing the mechanical and physical microenvironment needed by cells to proliferate and survive. Decellularized extracellular matrix (dECM) can be used as a powder, hydrogel and electrospun scaffolds. These bioscaffolds mimic the native microenvironment due to their structure similar to the original tissue. The aim of this review is to highlight the bone decellularization techniques. Herein we discuss: (1) bone structure; (2) properties of an ideal scaffold; (3) the potential of decellularized bone as bioscaffolds; (4) terminal sterilization of decellularized bone; (5) cell removing confirmation in decellularized tissues; and (6) post decellularization procedures. Finally, the improvement of bone formation by dECM and the immunogenicity aspect of using the decellularized bone matrix are presented, to illustrate how novel dECM-based materials can be used as bioscaffold in tissue engineering. A comprehensive understanding of tissue engineering may allow for better incorporation of therapeutic approaches in bone defects allowing for bone repair and regeneration.

## Introduction

Bone defects are generally caused by infections, tumors, trauma and degenerative diseases such as osteoarthritis, rheumatoid arthritis which cause major clinical problems and significant inability in patients worldwide [1]. It also has an overly impact on normal quality of life and health [2]. Subsequently, successful treatments are necessary and management of bone regeneration is essential. Although bone tissue can repair itself after injury, it is unlikely to repair itself whenever the severity of the bone damage is excessive [1]. Standardized treatment of bone defect in patients was based on bone grafts, which include autografts and allografts. Autografts are defined as the gold standard for bone regeneration, but both surgical procedures have drawbacks. The use of autograft, have some limitations and side effects such as restricted resource and donor site morbidity. Also autograft is not appropriate for reconstruction of large defects and have many risks such as pain, infection, and wound healing. Allografts have the same limitations in addition to the risk of immunological rejection [3–5]. Therefore, to avoid such limitations, innovative methods for repairing bone defects are essential. Bone tissue engineering provides an alternative treatment for bone defects [6] and develops organ transplantation without the need for allografts or autografts. In these methods, natural or synthetic bone substitutes can be transplanted in the patient. Over the past decade, researchers have focused on developing a suitable material as bone substitutes for bone replacement [7]. In regenerative medicine utilizing scaffolds and mesenchymal stem cells (MSCs), there is an excellent expectation for bone repair and regeneration. MSCs including Bone marrow-derived mesenchymal stem cells (BM-MSCs) and adipose-derived mesenchymal stem cells (AD-MSCs) are the main cell sources in bone tissue engineering (BTE). During recent years, AD-MSCs have attracted much attention as alternative sources of MSCs due to their ease of separation, widespread proliferation, and anti-immune nature [8–11]. dECM is a tissue-derived biomaterial that can be used as a natural component for tissue engineering applications. Decellularization is a process that removes cellular and immunogenic substances from tissues while preserving the natural component and mechanical properties of the ECM, which are critical for the delivery of oxygen and nutrients to the organ [12]. Therefore, designing innovative bioscaffolds that mimic the native environment is a strong interest in BTE. The dECM versatility allows it to be used for a variety of applications, including powder and hydrogel forms as scaffolds and digested solution as bioink for three dimensional (3D) printing. dECM scaffolds obtained from tissue decellularization have been applied as surgery mesh materials too. After Food and Drug Administration (FDA) approval, these materials were used in the clinics involving the intestinal herniation remedy, musculo -skeletal repair, sinew reconstruction, breast restoration, meninx fibrosa substitution [13] and gastro intestinal applications [14]. The present review article attempted to accumulate novel information in bone tissue engineering, specially the decellularization techniques to creat an ideal natural scaffold for effective bone regeneration. In this part of the review, we explain bone structure briefly before describing tissue engineering.

## The bone structure

**Fig. 1:**
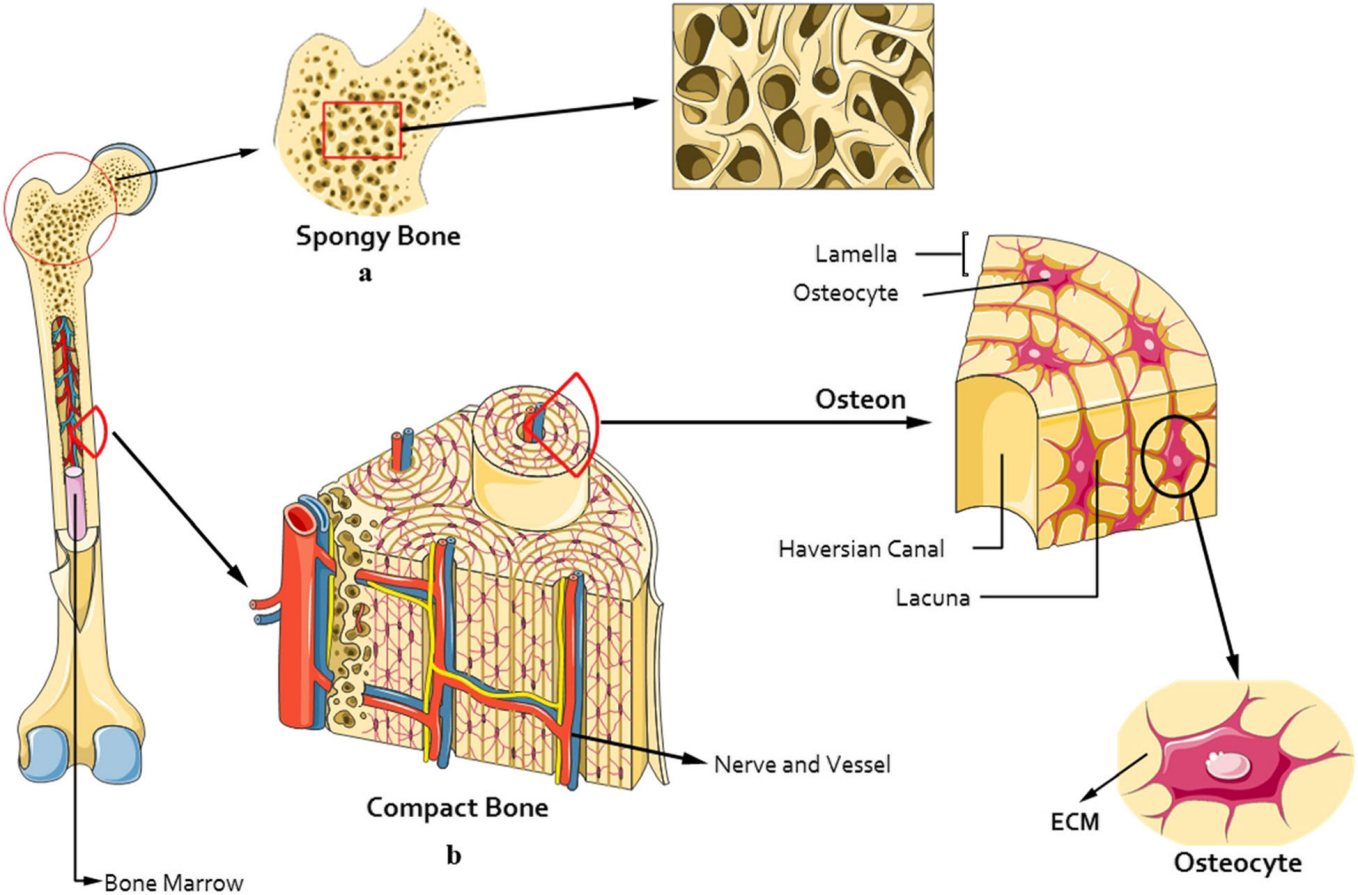
Explanation of bone structure. Two main types of bone include spongy (trabecular or cancellous) and compact (cortical) bone. a. The spongy bone is highly porous. b. The compact bone consist of osteon or haversian system, surrounded by the lamella which is made up of nerve and blood vessels

Bone is dense, calcified, and porous connective tissue [15]. Based on morphologic shape, there are three types of bone, including long, flat, and short bone. Based on structure, there are two main types of bone, including spongy and compact or cortical bone. Spongy bone*,* also called trabecular bone or cancellous bone. Spongy bone is lighter and less dense than compact bone. In spongy bone, the bone matrix is housed in a three 3D structure with spaces filled by the bone marrow. Compact bone forms the external layer of all bones and surrounds the bone marrow. It provides strength and protection to bones. Compact bone tissue is made up of units called osteons or haversian systems. The osteon, which is surrounded by layers of collagen, is called the lamella, which is made up of nerve and blood vessels (Fig. 1) [16].

### Bone extracellular matrix

Bone Extracellular matrix (bECM) is an essential noncellular constituents of bone tissue composed of type I collagen, glycoproteins, proteoglycans and noncollagenous proteins including osteocalcin (OCN), Osteopontin (OPN), osteonectin (ON), and sialoprotein [17, 18]. Osteoblasts play a critical role in the synthesis of bone ECM constituents. Growth factors are also found in bECM, such as fibroblast growth factor 23 (FGF23), bone morphogenetic protein growth factors / Transforming growth factor beta (BMPs / TGFβ) superfamily, fibroblast growth factors (FGFs), vascular endothelial growth factor (VEGFs) [19]. The complex nature of bECM provides a significant mechanical environment to simplify the specific function of bone tissue.

### Bone cells

**Fig. 2:**
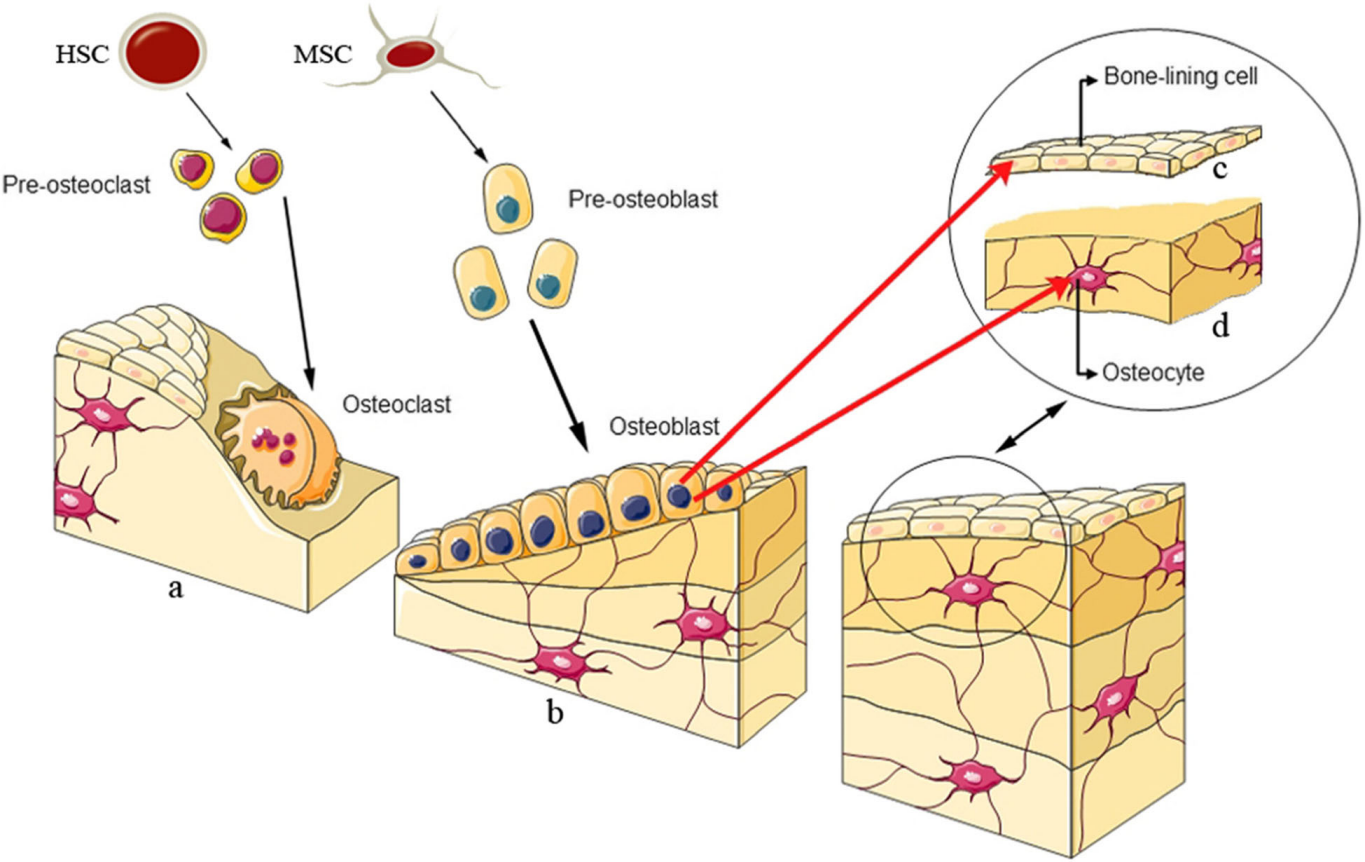
The picture portrayed different type of bone cells. **a**. Osteoclasts originate from hematopoietic stem cell, these cells destruct weary and ancient bone, **b**. Osteoblasts originate from mesenchymal stem cell that differentiates to the bone-lining cell and osteocyte **c**. Bone lining cell **d**. Osteocyte

There are four types of cells in bone tissue, including osteoblasts, osteoclasts, osteocytes, and bone lining cells. The structure is as follows as shown in Fig. 2. osteoblasts are bone-forming cells that play a critical role in a new bone generation. These cells originate from mesenchymal stem cells [20]. Various factors are involved in the differentiation of mesenchymal stem cells into osteoblasts, including Runt-related transcription factor 2 (Runx2), Distal-less homeobox 5 (Dlx5), Osterix (Osx), Bone morphogenetic proteins (BMPs), and members of the Wnt pathway [21–23]. The name “Wnt” is a combination of the wingless gene product of the drosophila and Int1, the protooncogene of mice. Osteoclasts originate from hematopoietic stem cells (HSCs). These cells destroy weary and ancient bone. In other words, osteoclasts are responsible for bone resorption; during this procedure, the bone mineral structure is broken down by enzymes released by osteoclast cells, while osteoblasts participate in new bone-forming regularly [24]. Osteocytes are long-lived dendritic cells that make up 90–95% of all bone cells. These cells originate from the osteoblasts in the lacunae, which are present in the calcified bone matrix. Osteocytes can reach the surface of bone and bone marrow to make connection with other osteocytes. Osteocytes are involved in bone remodeling by synchronizing with osteoblasts and osteoclasts in respons to even small bone deformities and mechanical stimuli. Bone lining cells (BLCs) are flat-shaped cells derived from mature osteoblasts. BLC, as a bone resorption, simplifies osteoclast function and participates in collagens digesting [25].

## Tissue engineering

**Fig. 3:**
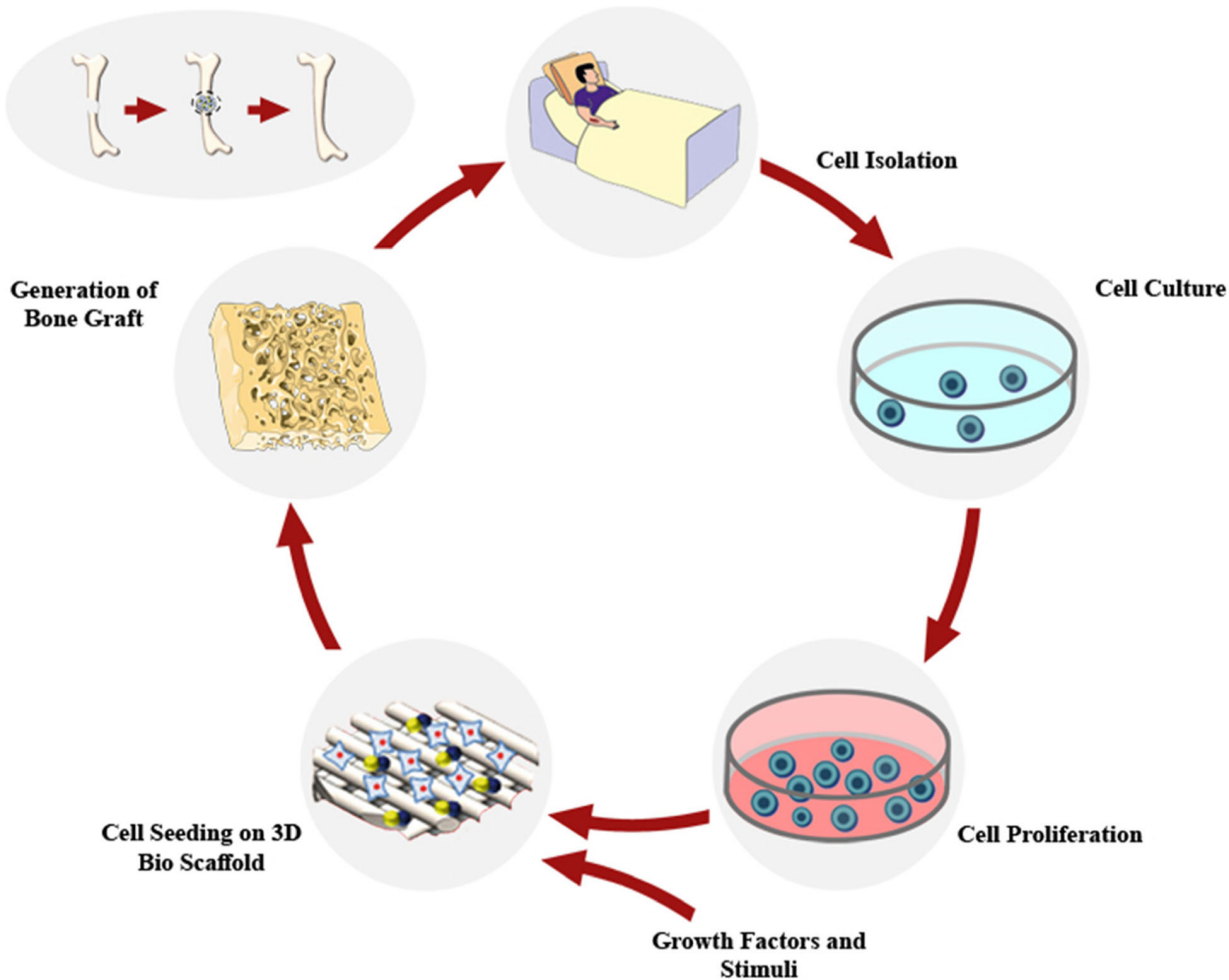
Schematic explanation of bone tissue engineering using 3D bioscaffold. Stem cells are derived from the patient. After seeding on 3D bioscaffolds, these cells differentiate into osteoblast and produce bone grafts to replace damaged tissue and organs

Tissue engineering is a multidisciplinary research field that applies stem cells, scaffolds, biochemical and biomechanical stimuli to make biomimetic biomaterials that fulfil the principal requirements for tissue creation in vitro or in vivo [26]. Stem cells are essential in regenerative medicine because of their significant potential for differentiate into various cell types as well as osteoblasts. AD-MSCs and human dental pulp mesenchymal stem cells (hDPMSCs) were commonly used in bone tissue engineering [27]. The use of an effective matrix containing stem cells supports a suitable environment for cell adherence, proliferation, and differentiation. The term “tissue engineering” was first presented in 1900 by Carell. In 1993, Langer and Vacanti designated tissue engineering (TE) as “biologic alternatives progression renovate, preserve and promote functional tissues” [28]. End-stage diseases and limited donors made a significant challenge in tissue engineering in the synthesis of tissues such as bone, skin, cartilage and bladder. In 1933 Bisceglie showed mouse neoplasmic cells enclosed in a polymer sheath and implanted in pig’s stomach, which was not rejected by the immune system. Furthermore, in 1975, Chick and his contemporaries in diabetics reported that glucose was regulated by pancreatic islet cells encapsulated by a semi-transparent membrane. Currently, there are skin reconstruction procedures that repair the skin using cells located in collagen, the history of this technique goes back to the 1980s [29]. Over the past two decades, considerable advances have been observed in tissue engineering [3] and regenerative medicine [1]. A significant goal in bone tissue engineering research is the use of various methods, novel 3D materials as scaffold, stem cells and biologically active molecules to make bone substitutes and grafts [30]. To construct a functional graft for bone regeneration and medical purposes, patient’s cells are obtained for the seeding on the scaffold to differentiate into osteoblasts, by which the immune system’s response is eliminated. Growth factors and stimuli are used to enhance bone regeneration. Hydrogel derived decellularized bone tissue can be used as a 3D bioscaffold. Hydrogels are suitable substrates for BTE due to their structural homogeneity, high permeability, biocompatibility and ability to be injected into the defect site. Tissue on this scaffold can be grown in vitro and then implanted at the defect site (Fig. 3).

## Scaffold considerations in tissue engineering

Scaffolds are one of the three crucial factors used in bone tissue engineering [6]. The word of scaffold is various synthetic and natural material that supplies the necessary foundation for conducting the inserted cells, stimulating cell attachment and cell reproduction in tissue and organ regeneration. Scaffolds also have a function as a transporter to growth factors and stimuli. Perfect scaffolds, should not collapse before new tissue is formed, except to provid an appropriate environment for cell proliferation. Additionally, they shouldn’t have immunogenic and toxic restrictions [31]. The main goal in scaffold designing is to create scaffolds that supply cell signaling and mimic the natural environment of tissues. Here we highlight the key features expected of bone scaffold engineering.

### Porosity and pore size

Some features such as porosity, size, and pore shape must be arranged according to the natural bone structure. Porous construction impacts the coefficient of elasticity and cell surviving. Porous construction supplies space, and position for cell proliferation, nutritional transport, and vascular growth [32]. Cell adhesion, and biomaterial deposition depend on pore-size and pore distribution [31]. The size of the scaffold pores should be big sufficiently to be effective in immigration but small adequately for efficient cell attachment [7, 33]. There are two kinds of pores according to their size; Macropore: The pore size of these scaffolds are bigger than 100 μm (micrometer). The existence of macropore in scaffolds simplify osteogenesis, angiogenesis, and support cell immigration to the implantation site. Some pores (150–800 μm) supply networks to vascular growth and nutritional transport that stimulate bone tissue regeneration [32]. Micropore: The pore size of these scaffolds are smaller than 10 μm. The existence of micropore in scaffolds play an essential role in osteoinductivity [34, 35]; for example supply particular texture that causes cell attachment. Naturally three dimensional (3D) bone structure have multi-scale pores such as nanopores, submicron pores, and micropores that make sites available for cell attachment [32].

### Surface properties

Scaffold surface properties include surface topography, smoothness, hardness, free energy, surface charging, and chemical properties. These features have an important impact on the scaffold’s ability to cell attachment and differentiation. Surface properties significantly are involved in the reactions between scaffolds and stem cells [36]. Also, surface property impact on viability and self-renewal of stem cells [37]. There are several methods to improve the properties of bone tissue scaffold, such as electro-spinning, sol-gel [38], three-dimensional printing, and solvent casting. Changing the surface based on the laser is an innovative technique that prevents inflammation. As scaffold porosity increases, protein uptake, permeability and degradation rate of biomaterials increase [39, 40].

### Biocompatibility

Biocompatibility is the more significant property of scaffolds, which supply a suitable environment for tissue regeneration without any adverse effects and inflammatory responses [32]. In other words, the scaffold and the host tissue are compatible with each other, so the immune response is not stimulated in this condition. The biocompatibility of designed scaffolds is usually estimated by cell binding and cell proliferation testing [30]. Decellularized bone scaffolds are appropriate biomaterials with high biocompatibility that are commonly utilized in biomedical applications [41].

### Biodegradability

In scaffold design, biodegradation is one of the characteristic that should be considered too. To maintain the structure and function of newly formed bone, the scaffold destruction ratio must be commensurate with the new bone growth ratio. This means that in the initial stage of tissue organization, the structure of the scaffolds must be sufficiently preserved and they must be dismantled after the reconstruction of the new tissue. Modification of biological materials of scaffolds based on radiation or oxidative reaction is an advanced technique that causes proper biodegradation [42].

### Mechanical properties and stability

Bone scaffolds provide the physical environment for new bone. It is very important that the scaffolds be stable and have mechanical properties similar to natural tissue [43]. The integrality of the scaffolds during implantation depends entirely on mechanical resistance. Mechanical resistance is usually determined by tensile and compression experiment tests [43].

### Osteoinductivity

The capability of scaffolds to stimulate the differentiation of stem cells into bone cells is called osteoinductivity [44]. In other words, osteoinductivity is a process in which osteogenesis is stimulated. This phenomenon generally occurs in the restoration of bone defects. Osteogenesis or ossification occurs in specific scaffolds in the absence of growth factors. The osteoinductivity feature of the scaffold stimulate stem cell differentiation and new bone tissue formation. Therefore, suitable bone scaffolds should have appropriate osteoinductive characteristics [45]. At the current time, the decellularized matrix are increasingly utilized, because these types of materials have significant potatial as bioscaffolds in the organization of bone tissue [46]. In outline, optimal scaffolds should have the characteristics described above.

## Extracellular matrix (ECM) as bioscaffold

Natural scaffolds derived from ECM, are broadly used in the preclinical research and clinical trials [47]. Medical meshes and wound care products made from ECM are safe and efficient. These bioscaffolds facilitate the effective reconstruction of several types of tissues as well as bone. ECM not only allows cells to be easily attached, but also affects cell migration, differentiation and proliferation [48]. The cell-derived extracellular matrix is a combination of collagen, water, fibrillary proteins, laminin, fibronectin, and macromolecules like proteoglycans and glycosaminoglycans (GAGs) [49]. Fibrillary proteins supply bonding sites for cells to attach matrix easily. Furthermore, these proteins arrange cell differentiation, migration, and gene - expression [50]. Laminin and fibronectin play an important role in the reaction between cells and the matrix [51]. The ECM provides the Arg-Gly-Asp (RGD) anchor tripeptide to simplify cell attachment. Furthermore, it affects the mechanical properties of the cell, which is an essential property for the transmission of mechanical signals. As a result, cells also affect the organization and mechanical properties of the ECM, cell interaction and ECM as a dynamic property of three - dimensional tissues is essential for optimal scaffolding [52]. These types of bioscaffolds are biodegradable and involved in regulation of cellular and homeostatic signaling. The tissue and organ decellularization technique provides dECM as a natural bioscaffold for tissue engineering purposes [53]. There are several decellularization methods for making dECM, that we will describe in this review.

## Decellularization

Decellularization is a technique that removes cells and immunogenic substances from tissues and organs [54], while preserving the natural ECM component [53]. Decellularized tissue matrix mimics the native microenvironment. It includes a specific organization and structure similar to the main tissue [55], which creates a suitable environment for cell function and differentiation [48]. The tissue decellularization technique was first used to treat burn patients in 1973 [56, 57]. In BTE, decellularized bone scaffolds are widely used because they have a 3D structure, high mechanical properties and osteoinductivity feature comparable to natural bone [58]. Decellularized bone matrix (DBM) include growth factor, fibronectin, heparin sulfate, chondroitin sulfate, and hyaluronic acid, which induce MSC differentiation into osteoblast. In addition, the porous nature of the bone, that affects differentiation, is preserved. Nevertheless, during all decellularization processes, some disruption of the ECM occurs. To produce ideal dECM scaffolds, a steady-state between maintaining the structure of the ECM and removing the cellular component is essential [59]. The main problem in decellularization is the elimination of all cellular compounds in the tissue without disrupting the ECM. Accurate and complete operation of this process is impossible, but it can be approached to some extent. Therefor efficient decellularization should maximize the withdrawal of cellular components and genetic material, while minimizing ECM disruption that preserves biological activity, three-dimensional ultrastructure, and specific biomechanical characteristics of the ECM [47].

## A common strategy for bone decellularization

There are several approaches to tissue and organ decellularization, such as physical, chemical, enzymatic, and perfusion decellularization techniques [52]. In bone tissue decellularization, the choice of bone type to create the scaffold is crucial. Between two types of bone matrix, spongy or cortical, spongy bone is suitable for decellularization due to its special architecture such as porous property and spongy construction. In addition, the surface–to-volume ratio of spongy bone is higher than that of cortical bone [24].

### Physical techniques

Physical techniques include the freeze-thaw cycles, supercritical carbon dioxide, and high hydrostatic pressure (Table 1). Physical processes such as pressure and temperature based methods are technically advanced. The use of these methods causes cell lysis, which destroys matrix proteins and eliminates genetic material [60]. In this section, we briefly describe these methods.

**Table 1:** Advantages and Disadvantages of physical decellularization

Technique		Mechanism	Advantages	Disadvantages	References
Physical decellularizationl	Freeze-thawing	Temperatures change alternately between − 80 °C and 37 °C. Liquid nitrogen creates ice crystals in the cell membran and destroys the cells.	No need for chemical reagents keeping the mechanical properties	Incomplete decellularization	[60]
	Supercritical carbondioxide (SC- CO2)	At a pressure of 30 MPa and a temperature of 50 °C, cells and genetic material are removed from the bone tissue.	High biocompatible No need for terminal sterilization Preservation of ECM construction Perfect decellularization Fast Nontoxic	No disadvantages have been reported for this method.	[61]
	High hydrostatic pressure (HHP)	Disrupts cell membrane through high hydrostatic pressure	High biocompatible No need for terminal sterilization Preservation of ECM construction Perfect decellularization Fast	No disadvantages have been reported for this method.	[62]

#### Freeze-thawing

The freeze-thaw method usually includes changing the temperature between − 80 °C to 37 °C, respectively. Usually two to three freeze-thaw cycles are required for effective tissue decellularization. The use of liquid nitrogen creates ice crystals in the cells that penetrate the cell membrane and destroy the cells. The advantages of using this method are the reduction of chemical use and preservation of ECM ultrastructure [60].

#### Supercritical carbon dioxide (SC-CO_2_)

Supercritical carbon dioxide (SC-CO_2_) is used to tissue decellularization due to its compatibility with biological materials and also leaves no toxins in the scaffolds. SC-CO_2_ has been shown to remove cells and genetic materials from bone tissue at a pressure of 30 MPa (megapascal) and a temperature of 50 °C. For efficient decellularization, rapid pressure reduction is necessary. High pressure causes the cells to spurt and push the cells out of the tissue. SC-CO_2_ processing was performed with Speed SFE 4 system (Applied Separations, Allentown, PA). The method of using decellularization on SC-CO_2_ eliminates the need for final sterilization and does not cause structural and mechanical changes in the scaffold. Furthermore, the use of this method causes perfect decellularization and decreases processing time [61].

#### High hydrostatic pressure

High hydrostatic pressure (HHP) is a physical decellularization technique that destroys the cell membrane by increasing hydrostatic pressure. In this way, the structure of the tissue is significantly protected, while the genetic material and the nucleus are completely eliminated. HHP kills viruses beside decellularization, therefore, the sterilization process is no longer necessary. In addition, it does not contain chemical reagents, so by eliminating the reagent, the need for washing is excluded. Pressing the bone tissue using a static cold pressure machine (Chef: Kobe Steel, Kobe, Japan) was performed at 1000 MPa and 10 °C for 10 min. Under these conditions, the cell membrane ruptures with increasing static pressure [62]. Also biocompatible scaffolds with the ability to regenerate bone are obtained using this method [63].

### Chemical decellularization

There are three divisions of chemical decellularization protocols: detergents, acidic or basic conditions, and chelating agent (Table 2) [64, 65].

**Table 2:** Advantages and Disadvantages of chemical decellularization

Technique		Mechanism	Advantages	Disadvantages	References
chemical decellularizationl	Ionic Detergent	SDS eliminate nuclei, DNA and breaks up protein-protein bonds. Sodium deoxycholate dissolves nuclear and cytoplasmic membrane.	Highly effective	Damages ECM structure and GAG Reduces growth factors	[66]
	Non-ionic Detergents	Triton x-100 dissolves proteins. It destroys cell membrane and cell lipids. Triton x-100 breaks down lipid-lipid, lipid-protein and protein-DNA bonds.	Biodegradable Perfect detergent	Damages collagen and GAGs	[66]
	Acids Bases	break down nucleic acids, and hydrolyze cytoplasmic components *Solubilize cell membrane and cytoplasmic components*	No advantages have been reported for this method	Acids damage ECM structure and reduce GAGs Bases significantly reduce GAGs and reduce the mechanical properties of ECM.	[60]
	Chelating Agent	EDTA binds to metal and causes cell separation.	No advantages have been reported for this method.	Prolonged use of EDTA reduces the mechanical properties of the scaffold	[67]

#### Ionic detergent

Sodium dodecyl sulfate (SDS) is a synthetic organic compound with the formula CH_3_(CH_2_)_11_SO_4_Na. It is an anionic surfactant used in many cleaning and hygiene products. SDS is used as an ionic detergent to destroy cell nuclei [68] and to remove deoxyribonucleic acid (DNA) in a variety of tissues and organs such as bone. Compared to other detergents, SDS is very effective in removing cytoplasmic compounds and cell debris [65]. So SDS are widely used to destroy genetic material, proteins and collagen [62] in different types of tissues and organs. SDS breaks the bond between proteins and dissolves the cell membrane that leads to cell destruction. But we must consider a higher concentration of SDS damage to the cell structure [64]. Also, the use of SDS has drawbacks, including reduced glycosaminoglycan content and ECM growth factor. Another example of an ionic detergent used in the decellularization method is sodium deoxycholate and triton x-200 [69, 70].. Sodium deoxycholate is less commonly used because it has an additional destructive effect on cells compared to SDS [71].

#### Non-ionic detergents

Triton x-100 is a non-ionic surfactant and biodegradable emulsifier can be used in biochemical applications to dissolve proteins and degrade cell membranes [64]. Also, compared to most detergents and even lipase, it is a complete detergent in the lipid removal process. This detergent destroys cells and ECM glycosaminoglycans, thus creating a natural environment for cell growth. Triton x-100 breaks down lipid- protein and protein-DNA bonds [72].

#### Acid and base

Acids and bases work to tissue decellularization, break down nucleic acids, and hydrolyze cytoplasmic components. Acids destroy collagen, GAGs and ECM growth factors. As a result, an interesting topic is optimizing the amount and percentage of acids used. The success rate of decellularization will depend on the type, acid/base density, and duration of treatment [73]. Acetic acid, hydrochloric acid, sulfuric acid and peracetic acid (PAA) are the most common acids used in the decellularization method. The most commonly used bases are sodium hydroxide, sodium sulfide and calcium hydroxide [65]. As mentioned above, during decellularization, GAG maintenance is essential to maintain the biomechanical structure of the ECM. Because bases significantly reduce the amount of GAG, the bases are not used during bone decellularization [73].

#### Chelating agents

Such compounds as ethylene diamine tetraacetic acid (EDTA) and ethylene glycol tetraacetic acid (EGTA) are utilized in the decellularization process. Chelating agents bind to metal ions such as Ca^2+^ and Mg^2+^, and cause cell separation [47]. EDTA is commonly used in combination with trypsin and other enzymes. For example, in a protocol proposed by Later, Sladkova, and colleague [74], EDTA was utilized in combination with deoxyribonuclease (DNase), ribonuclease (RNase), and detergents such as SDS to remove cell debris from human bone [67]. Moreover, long-term use of EDTA decreases the mechanical properties of the scaffold [65]. Therefore, we must be careful about the duration of use of this substance.

### Enzymatic decellularization

To remove cell and nucleus residues, enzymatic decellularization method following chemical agents is necessary (Table 3). Common enzymes used in this method are proteases and nucleases. Proteases, for instance, trypsin, a proteolytic enzyme commonly used with EDTA, breaks down cell-matrix adhesions and hydrolyzes proteins through chain cleavage in lysine or arginine residues. After that, ECM proteins such as collagen and elastin are destroyed. The duration of treatment should be reduced to minimize the damaging effects of the enzyme. Nucleases, including RNases and DNases, break down nucleic acid sequences that cause nucleotides to be lost during cell lysis [76, 77]. In general, the physical method does the least damage to the cells. On the other hand, the destruction of cellular material would not be effective without the use of chemical methods. Similarly, in chemical treatments without physical processes, effective decellularization does not occur due to limited dispersion of substances in cells. Physical methods make it possible for chemicals to penetrate cells more quickly and easily. As a result, due to the synergistic effect of all three methods decellularization protocols are usually a combination of physical, chemical, and enzymatic strategies [73].

**Table 3:** Advantages and Disadvantages of enzymatic decellularization

Technique	Mechanism	Advantages	Disadvantages	Reference
Enzymatic Decellularization	Proteases: trypsin: Breaks down cellular proteins on the c-side of Arg or Lys and then destroys ECM proteins such as collagen and elastin. Pepsine: Breaks the bounds between peptides Nucleases: Break sequences of nucleic acids.	highly effective	Can damage the proteins in the ECM, especially laminin and GAG It changes the structure of the matrix Further cleaning and enzyme removal is required They may promote immune response.	[75]

### Perfusion decellularization

There is an advanced technology to improve the quality of decellularization that can balance the stability of the ECM structure with the elimination of cell contents. In perfusion decellularization, the structure of the multiplex and the complete vascular template of different tissues and organs are preserved. The predominant goal of this technique is to create an ideal acellular matrix in which the three-dimensional structure and ECM proteins are protected [78]. This biotechnological method can be used to create ECM scaffolds in medical applications and clinical studies such as porcine urethral decellularization to create decellularized scaffolds for tissue engineering applications [78]. Perfusion decellularization has already been performed on complex organs such as lungs, heart, kidneys and whole liver [79]. Some composite bioscaffolds are achieved using the perfusion decellularization technique, which is a fundamental platform to construct tissue for ex-vivo and in vivo investigation and clinical trials [36].

#### Bioreactor for perfusion decellularization

A perfusion decellularization bioreactor made from a propylene box is used to whole-organ decellularization. It is equipped with running containers (Nalgene; 2319–130) and sterile connectors (Cole Parmer). Both box and solution containers are equipped with high-efficiency particulate air (HEPA) filters to provide aeration while keeping sterile (Whatman; L # 9514261). The top of the box is enclosed using a silicone seal and a polycarbonate cap (McMaster-Carr; 86,045 K23, 8574 K55). Every thing is done in a multi-layer flow hood. Perfusion pressure is controlled via a disposable linear pressure sensor (Pendotec-Pressure MAT) [36].

## Terminal sterilization of decellularized bone

Terminal sterilization of decellularized bone matrix is necessary to make them safe before medical applications. The goal of terminal sterilization is mainly to eliminate the genetic material of microorganisms such as bacteria, viruses, and fungi. There are four important sterilization methods include ethylene oxide exposure, gamma irradiation, supercritical carbon dioxide, and electron beam irradiation [80]. Electron beam irradiation and gamma irradiation are commonly utilized, but they have negative impact on the mechanical strength of DBM. Peracetic acid (PAA) is another alternative method that kills bacteria, fungi, viruses, and spore from scaffolds, while not changing mechanical properties of the scaffold and protect the structural proteins of the scaffold. Supercritical carbon dioxide (SC-CO_2_) in combination with co-solvents, such as tert-butyl hydroperoxide and PAA can eliminate bacteria and viruses without any side effects on the ECM structure compared to other methods. This method is superlative in achieving effective sterilization [81]. The advantages and disadvantages of these methods are outlined briefly in Table 4. Some methods have side effects on the structure of ECM especially on hydrogel form of these scaffolds. Therefore it is important to indicate the appropriate method, because some methods such as sterilization by acids and any solution, cause the principal destruction of the structure of the ECM. The perfect method should exclude all scaffold contaminations without substantial damage to the biomechanical properties of the scaffolds [83].

**Table 4:** Advantages and Disadvantages of terminal sterilization methods

Sterilization techniques	Advantages	Disadvantages	References
Gamma Irradiation	Fast Safe	Alters the mechanical strength of bioscaffolds	[81]
Electron Beam Irradiation	High biocompatible Fast Safe	Alters the mechanical strength of bioscaffolds Damages the ECM architecture	[80]
Ethylene Oxide	Has no effect on the ECM ‘s ability to bind T- cells. Has no effect on the secretion of growth factors on fibroblasts.	It alters the structure of the protein, making it impossible to trace these molecules. Changes the mechanical stability of the ECM. Mutagenic Carcinogenic It may promote immune response	[82]
Supercritical Carbon Dioxide (principal method for terminal sterilization)	A practical method High biocompatible keeping the mechanical properties Preservation of scaffold stability Safe	No disadvantages have been reported for this method	[47, 81]

### Cell removing confirmation in decellularized tissues

The common gold standard method for evaluating effective decellularization is to determine the total amount of DNA in sampled tissues before and after decellularization. As stated by Crapo et al. at least three principles must be considered to confirm effective decellularization. First, One milligram of dehydrated decellularized tissues ought to contain less than 50 nanograms of double stranded DNA (dsDNA). Second, all remaining DNA fractions must be less than 200 base - pairs. Third, decellularized samples should not have a clear nucleus in hematoxylin and eosin staining as well as dapi staining. In addition to assessing cell content, biochemical evaluation of ECM is also essential. In the case of whole tissue decellularization, imagining techniques, for example scanning/transfer electron microscopy (SEM/TEM) and micro – computing tomographic imaging can be utilized to compare the structure of the ECM including before and after decellularization [84].

## Post-decellularization procedures

**Fig. 4:**
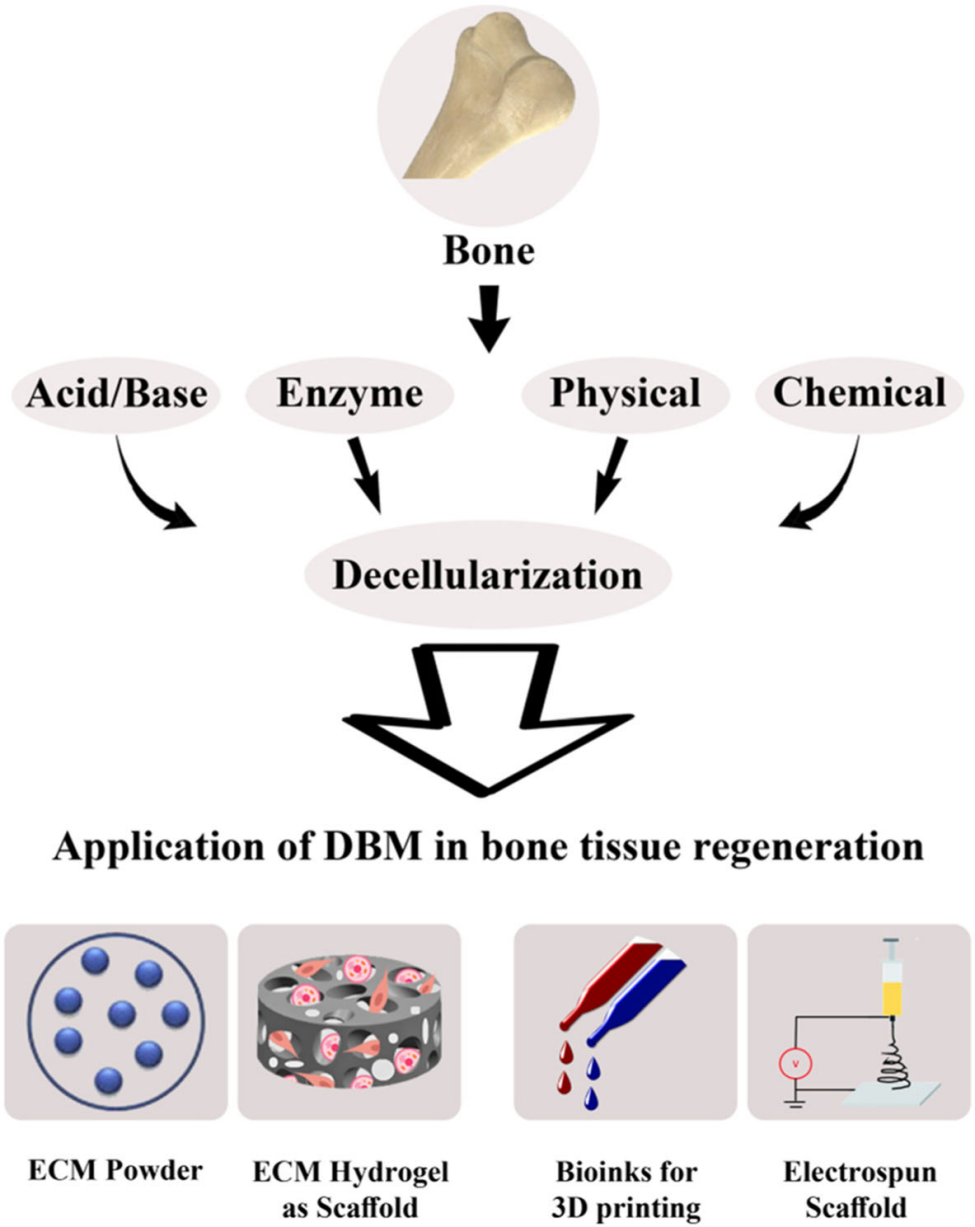
Schematic explanation of DBM-derived bone tissue that can be used as a powder, ECM hydrogel, bioink and electrospun scaffold in bone tissue regeneration

In general, DBM can be used as hydrogel, bioink and electrospun scaffold beside powder form after decellularization to meet various requirements (Fig. 4). The Post*-*decellularization procedure substantially enhances the efficiency of decellularized bone. Here we propose some post*-*decellularization procedure that improve the properties of dECM and overcome some deficiencies, such as poor mechanical strength and weak bioactivity that occur during bone decellularization [85].

### ECM hydrogels

The term “ECM hydrogels” is utilized for hydrogels made from decellularized mammalian tissues such as bone. The fabrication of ECM hydrogels consists of three main steps: 1) Dissolution of dECM with pepsin into protein monomer components; 2) pH neutralization to stimulate the modification of the intra-molecular bonds of the monomer composition to a homogeneous gel; and 3) hydrogel formation with increasing temperature to 37 ° C. ECM hydrogels have been used effectively in BTE due to their balance of biochemical and physical features. These scaffolds are multipurpose resources and have a variety of potential applications, including 2D and 3D scaffolds, where cells can be cultured on or inside the hydrogel. ECM hydrogels are also used as an injectable material in bone regeneration due to their higher water content and softness [86]. Collagen is the main structural protein of dECM, which plays an important role in maintaining the biocompatibility and biodegradability of ECM hydrogels. Hydrogels also have elastic properties comparable to natural tissues. So they stimulate bone regeneration, create a safe environment against infection, control inflammation and eliminate wound secretions. Even if hydrogls have clear advantages, introducing these new biological materials to the market and clinics is challenging due to the difficulty of mass manuifacturing and low clinical trial studies.

### Decellularized extracellular matrix as bioink

The introduction of 3D bioprinting technique has a great impact on the progress of bone regeneration. There are various methods of bone bioprinting including inkjet, light based 3D and extrusion. These modern techniques may improve osteoinduction and osteoconduction in bone regeneration. As a result, it can lead to the production of effective bone substitutes such as allografts or xenografts. In this technique, basic factor such as bioink is essential for bioprinting. Bioink is an ECM-like substances that contains the internally enclosed cells that make up the scaffold. Bioinks have been crucial in the development of 3D bioprinting for bone tissue engineering in recent years. A biomaterial such as soluble dECM is a gel-like biomaterial appropriate for 3D bioprinting. dECM as an intermediate product is generally used for the production of 3D structures. In this process, hydroxyl apatite (HA) or /β-tricalcium phosphate (β-TCP) nanoparticles (60–80 nm) can be dispersed in hydrogel-based bioink to enhance osteogenesis. Nanoparticles intensely impact cell behavior and perform better like a natural ECM. HA-released ions stimulate stem cell differentiation (osteoinduction). Other nanoparticles such as gold, magnetic iron oxide and molybdenum-doped bioactive glass displayed osteoinductive capabilities too [87]. In this method, Bone surpasses other tissues in terms of clinical application. For example, bioprinted bone is effectively implanted in pre-clinical models. In addition, 3D printing implants are effectively implanted as xenografts to replace bone tissue in humans. In such fields, advanced technology is increasingly highlighting the need for laboratories with multi-disciplinary skills. Further, the establishment of standard regulatory conventions is essential. The most important of them is the increasing need for translation into medical applications of these products [73, 88].

### dECM -based electrospun nanofibrous scaffolds for bon regeneration

Electrospinning as a method to create fibrous scaffolds is a manufacturing technique involving an electrostatic derivative process used to create electrospun fibers. Nanostructured scaffolds are considered as supreme tissue engineering scaffolds for their complex interface topology,wide surface area, and ease of functionalization. Nevertheless, application of electrospun scaffold obtained with conventional electrospinning techniques due to the provision of 2D nanostructured morphology, penetration and migration of cells on the electrospun matrix are limited. Many studies have revealed that the advanced manufacturing of biomimetic electrospun mats is a crucial aspect of a successful regenerative process. In recent years, the research in scaffold engineering has shifted from using natural polymers to using the dECM to achieve scaffolds mimicking native ECM. The application of dECM products supplies an ideal microenvironment for cells, with sufficient chemical and biological signs essential to regulate behavior of cells. In vitro testing displayed that the aligned nanofibers of decellularized muscle tissue and polycaprolactone polycaprolactone (PCL) supports satellite cell growth, myogenic protein expression, and myokine production [73].

## The decellularized tissue mimics the native microenvironment

The Native microenvironment (NME) is the local microenvironment of cells or tissues that provides them mechanophysiological condition [89]. NME govern a cell function, for instance, proliferation, differentiation, migration and secretion of bioactive molecules. Specifically, cells control their NME and the NME govern the function of cells. Commonly, tissue specific stem cells, MSCs, fibroblasts, endothelial cells, immune cells, adipocytes, lymphatic cells, and migrating cells take part in the formation of NME [90, 91]. The main goal in tissue engineering is to mimic a native- like microenvironment in in vitro system. Therefore, tissue-specific and biocompatible dECMs are very hopeful for regenerative medicine and tissue engineering, and can be used without the limitations from the lack of donor organs or tissues [89]. Studies have shown that natural derived biomaterials such as dECM, ECM and silk fibroin are the most important components for the homeostasis, growth, and repair of tissues [92]. dECM scaffolds can well mimic the composition, distribution, and biochemical signals of various matrix components of native bone tissue [49]. Integration the structure and function of tissue engineering scaffolds is great important in mimicking native bone tissue. As mentioned before, Hydrogel derived dECM can be used as a native 3D scaffold that mimics an optimal environment with various bioactive components. dECM contains approximately all the ECM proteins that play an important role in the conformation of native- like microenvironment [93]. The decellularized bone matrix maintains the macrostructural topographies such as highly porous structure and geometry and microstructural characteristic such as micropores and surface roughness, which significantly increases the osteoinductivity. Research has shown that cell behavior varies in different dECMs, with specific dECMs selected for specific tissue engineering, for instance bone dECM for bone regeneration. In addition, their environmental conditions and mechanical performance are similar to those of native ECMs. Native ECM includes memory cues and factors that maintain definite tissue memory that can differentiate specific tissue, for example, rigid decellularized bone matrixes mimic the cross-connected collagen structure of osteoid, thus stem cells on rigid substrates have a tendency to differentiate into osteogenic cells [94]. dECM scaffolds not only maintain immunomodulatory cytokines such as TGFβ and BMPs that control the pro-inflammatory response, but also optimally provide cell-matrix and cell-cell interactions through native-mimicking signaling events [49]. Duo to the limitations of the decellularization technique, for example, changes in the chemical composition and physical configuration of ECM proteins during the decellularization process, which destroy native topographic properties and active sites, ECM tissue must be carefully decellularized to produced dECM exactly like native ECM and contains all native tissue proteins. Thus, despite the full understanding that dECM is an exellent alternative, there is still the challenge of creating a native- like microenvironment using these biomaterials [89]. In general, both natural and synthetic materials should not only supply mechanical, structural, and biochemical supports for cells cultured within the scaffolding materials, but should also optimally aid cell-cell and cell-matrix connections via native-mimicking signaling processes.

## Summarised in vivo studies in bone defect animal model

To achieve effective DBM-derived bioscaffolds in BTE, animal bone defect model studies are crucial. Animal model studies have provided important data and knowledge with the aim of developing expected and ideal bioscaffolds. The most common models of bone defects used in animals are mice and rabbits*.* In the case of mice, the investigation of skull and femur bone defects has received more attention than other parts of the body. In contrast, fewer studies have been performed on mandible and fibular. Significant studies have shown that decellularized bone grafts have excellent regenerative effect on bone defects [41]. For instance, Min Suk lee. et al. have made the DBM-derived bioscaffolds that can be utilized in medicine and bone regeneration. These scaffolds contain bone-forming active biomolecules and genes involved in bone growth, as well as a special structure that mimics the natural environment of the ECM [84] In an experiment by Onishi et al., an osteogenic ECM sheets, that preserve the growth factors and native collagen I was used as a bone graft in a femoral defect in a rat model, implantation of this dECM scaffold increased bone regeneration [95]. In rabbits, the radial bone defect model has been studied more than the cranium and femoral bone defect models. Other models of rabbit bone defects such as femoris, tibiofibular, cubit, crania, mandibula, humerus posterolateral were also studied. It is also necessary to study larger animals that are more similar to the physical properties of human bone. In large animals, including pigs, dogs [96, 97], sheep [98], and horses [99] the bone defect model has been investigated to evaluate the ability of decellularized bone grafts to repair defective bone. Overall, published scientific works have shown that research on bone decellularization studies is less common than organs such as the lung, pancreas, cartilage, and heart.

## Infrequent clinical trial of bioscaffolds in the human body

Decellularized bone bioscaffolds facilitate bone repair in clinical applications. Karalashvili et al. showed that decellularized bovine femoral bone is used to generate 3D bioscaffolds. These bioscaffolds were used to reconstruct zygomatic bone defects in patients with car accidents. The practice was carried out under the ethics rules of the 1975 Helsinki Recommendation and authorized by the S. Khechinashvili University Clinic, Tbilisi, Georgia Ethics commits. Decellularized bovine bone matrix was used as a scaffold to create xenografts for bone tissue generation purposes, such as maxilla- facial bone regeneration. In a clinical study by Ann kakabadze et al., decellularized xenograft-derived scaffolds were used as bone graft substitutes in a patient who endured principal mandible tumor and hemimandibulectomy. This graft had outstanding and appealing outcome. In the other three patients under the same condition, the mandibular defect was repaired by autogenous rib graft. All four patients were examined twice a year after mandibular repair, none of whome had any particular problems [100]. In an experiment like this, decellularized xenograft-derived bovine bone scaffolds was used as a bone graft in a tibial defect in a 58-year-old woman. Six weeks after implantation, newly formed bone was identified and the patient was able to walk comfortably [101]. Furthermore, decellularized bovin bone bioscaffolds with patient autogenous MSCs were used in the clinical treatment of distal tibia reconstruction. Six months after treatment, active and new bone formation was diagnosed in the patient. This clinical trial demonstrate that DBM implantation affect cortical bone repair beside spongy bone repair [102]. Overall, the results of these clinical trials indicate that xenograft-derived bone scaffolds can be used as an alternative to autologous bone grafts although supplementary studies are require. FDA-approved decellularized bone-derived products including Puros® DBM, BioSet™, Grafton®, DBX®, Progenix™ Plus, Accell Connexus® & TBM®, InterGro®, Viagraf®, have been used in clinical applications such as bone and tendon repair [85]. Based on the myriad of articles, we can state the fact that decellularized bone grafts can be used for major reconstruction of bone defects.

## Improvement of bone formation by dECM

The dECM contains a tissue-specific carbohydrates and bioactive proteins. Some functional epitopes of the proteins, after embedding, organize bone-specific physiological and biochemical signals into the attached cells and bound to cytokines and growth factors that are naturally retained in the bone matrix to guide bone formation [103]. In addition dECM provide, cell-matrix and cell-cell interactions could cause the gradual integration into the host tissue during the healing process. Recent studies showed that the decellularized ECM and the ECM secreted by MSCs play important roles in bone regeneration, the ECM secreted by MSCs could be the bridge for cell attachment and it causes MSCs homing [104]. Furthermore, they can create the microenvironment to maintain homeostasis and they also can enhance the survival capacity of MSCs effectively [49, 102].. In a study, dECM hydrogel implanted in rat femoral defects considerably enhanced osteopontin and collagen expression and regenerated large volumes of bone after 6 weeks compared with controls. dECM contains laminin isoforms, collagen type IV, proteoglycans and heparan sulfate. Surface-exposed epitopes of these proteins and carbohydrates can produce direct signals by binding integrins to bone marrow mesenchymal stem cells (BMSCs), osteoblasts, and osteoprogenitors. Responding to this signal, osteogenic cells can release TGFβ and VEGF that activate migration of cells into the defect and vascularization respectively. Bone marrow endothelial progenitor cells, in response to VEGF, can secrete BMPs 2,4 and 7, which cause osteoprogenitor migration, differentiation, and thus initiate a positive feedback looStimulation of these progenitor cells by binding to integrins can also lead to the secretion of TGFβ and insulin-like growth factor (IGF) [105].. These results imply that the presence of marrow is important for cortical bone healing. In the study of Taylor et al. a decellularized cortical bone scaffold was introduced that imitates the cylindrical construction of native cortical bone and contains biological markers without the use of growth factors to stimulate endothelial growth and stem cell differentiation along with angiogenesis. This novel bone technology has the potential to promote bone regeneration in large bone defects [106]. Junka et al. studied an effective technique for fabrication of dual-layer polymer-dECM scaffolds with the aim of enhanced bone formation in femoral cortical bone defect. They showed that implanted scaffolds improved bone growth in femoral cortical deficiencies, and constructs with both osteogenic and vascular cues considerably amended cortical width [107]. During bone regeneration, MSCs homing, osteoid mineralization, osteoblasts formation and osteocytes differentiation all act significant roles in bone formation. The perfect bdECM supports specific biomechanical environment, bone-specific physiological and biochemical signals that influences the adhesion, proliferation, and cell fate decision [108]. More importantly, the regenerative outcome of MSCs and dECM in animals is related to their immunoregulatory capability, which can promote the polarization of the macrophage response from an inflammation and tissue injury process (M1 phenotype) to a regeneration process (M2 phenotype), which is a promising strategy for injuried tissue repair. But the exact mechanism especially, the interaction between dECM scaffold and MSCs, macrophages—is indefinite. Furthermore, the interactions of ECM secreted by MSCs and the decellularized ECM scaffold need to be investigated.

### Immunogenicity aspect of using the decellularized bovine bone matrix

Most important and basic issue in fabrication decellularized bone matrix scaffolds is how to completely remove the cell contents. Immunological responses can be stimulated against cells, DNA, lipid or galactose-α1,3-galactosyl-β,4-Nacetylglucosamine (α-Gal) epitope of incomplete decellularized scaffold implants. Immunogenicity and cytotoxicity of xenogenic ECM scaffolds are a limitation of these scaffolds in clinical application [67]. Thus, our eyes are focused on an effective decellularization that eliminates cellular content and DNA, as this DNA activates immune responses by stimulating B cell immunoglobulin production and cytokine secretion after implantation. In addition, residual DNA can stimulate the M1-type macrophage response in the host-implanted area, and Gal epitope which is expressed on cell-surfaces in non-primate mammals, including cows and pigs, lead to an immune response in the human body [84]. You et al. have reported that using a SC-CO2-based decellularization technique could achieve a highly immunocompatible decellularized scaffold. They systematically investigated the in vivo immune responses such as the spleen index as an important immune organ, histology, cytokine, in vitro splenocyte proliferative performance, immune cells contents and immunoglobulin light chain expression after transplantation decellularized bovine bone matrix in mice. The experimental results displayed that the immune responses of decellularized group in comparison with native bone group were significantly decreased [109]. In another study by Ling et al. it was found that using SC-CO2-decellularization method, remove 100% of Gal epitopes [110]. These studies recommend the use of SC-CO2-based decellularization technique to produce biocompatible scaffolds. Also, in the research by zhang et al. evaluation of the immune response to bovine collagen was assessed in a mouse model. The results indicated that the morphology of the spleen and lymph nodes did not show obvious swelling in mice following different amounts of collagen implantation. Furthermore serum IgG and IgM concentration, the CD4/CD8 cell ratio in lymph nodes and spleen was almost normal following collagen implantation [111]. It can be said that by using the effective decellularization technique, a highly immunocompatible decellularized scaffold can be achieved.

### Large-scale and commercial applications of dECM

The scale-up process is one of the most challenging process of technology transfer from the laboratory to the industrial scale. Scale-up attempts to obtain products on a large scale in the laboratory with the same performance and features. Likewise, additional factors, such as process control or its reproducibility, must be considered. The whole process need to be optimized, both engineeringly and economically [112]. There are substantial challenges inhibiting the scaling up the decellularization. One of the related issues is the great deviation between different decellularization techniques. As decellularization technique is a relatively new laboratory process, related devices and technologies are predominantly non-standard and only available in small laboratory-specific scale. Some decellularization techniques are used to increase the scale, efficiency, or automation of the process. Commercial companies that have industrialized ECM-based biomaterials, focusing on decellularization methods and processes rather than advancing specific automated decellularization systems. Commercial companies such as Langerdorff and Miromatrix adapted different perfusion devices. Miromatrix creates the whole organ dECM for recellularization, although Langendorff concentrated on its method for utilize in physiological and pharmacological studies. Xylyx Bio is another commercial companies that creates porcine and human-based ECM, but does not disclose the methods and strategies it uses. There are a few devices that have been settled by companies such as Ebers and Harvard Apparatus (HA) for organ decellularization. Harvard Apparatus developed the perfusion-based Bioreactor in 2013. It has several chambers with several sensors for checking the processes, such as pH, pressure and flow meters. This device is one of the few commercially available bioreactors [113]. Additionally, Perfusion-based decellularization systems such as the Large throughput system with 200-l tanks and the automated decellularization technique increase both the scale and efficiency of the decellularization technique which, produces a higher volumes of dECM. The large throughput system contains 200 L tanks filled with reagents for decellularization. The tanks can be connected to 24 perfusion lines allow several organs to be decellularized in parallel. In this decellularization system, each organ is independent of each other. There is also a shortage of specific decellularization bioreactors and devices in the commercial market, which is probably due to the beginning of this field. Research teams are working to standardize decellularization methods, develop more automated systems and ultimately create the ideal decellularization system that can create compelet dECM with maximum efficiency. An additional prospective area for researching and improving this system is the combination of different protocols alongside a simplified protocol to increase performance and quality while reducing human operation and duration [114]. Kusuma et al. produced MSC-derived dECMs on 2D tissue culture plates and processed the dECM to generate a concentrated biomolecule solution that could be utilized to cover new surfaces, while maintaining optimal bioactivity, would significantly enhance the practical application of these materials. These transferable matrixes can aid scale-up of this technology by enabling the production of higher cell culture surface with the predicted bioactive properties of native dECM and allowing more surface coverage compared to native dECM [115]. The functions of cultured cell-derived decellularized matrixes is changed by many factors such as cell-to-cell variations for example, the types of cell lines, gender, passage number and age [116]. In addition, decellularization methods and storage methods affect the quality of the final product, that have not yet been optimized. Although dECM scaffolds are commonly catogerized in medical devices, there are still no legally defined or accepted standards for quality control of these matrixes for medical applications [114].

### Conclusion and prospects

The most common technique for repairing large bone defects is surgery and bone grafting. Although autografts are defined as the gold standard for bone repair, there are various weaknesses with autografts, such as restricted resources and donor site morbidities. With innovative findings and further improvements in tissue engineering, DBM as bioscaffold could be used for bone defect healing. At present, fabrication of bioscaffolds by decellularization techniques is an effective strategy in tissue engineering. As the text exhibits, bioscaffolds are involved in regulating cellular signaling and tissue homeostasis. Fibrillary proteins of native ECM supply bonding sites for cells to attach scaffold easily. The particular feature of these bioscaffolds to induce bone regeneration is due to the similarity of these scaffolds with the native bone structure. dECM scaffolds can well mimic the composition, distribution, and biochemical signals of various matrix components in native bone tissue. The perfect bdECM supports specific biomechanical environment and biochemical signals that influences the adhesion, proliferation, and cell fate decision. However, there are some limitations in preparing bioscaffolds, including the protection of ECM ultrastructure and biological signals during the decellularization procedure. Also immunological responses can be stimulated against cells, DNA and α-Gal epitope of incomplete decellularized scaffold implants. Additionally, there are other limitations to the conclusion about the ideal decellularization method. For this purpose and clinical use of DBM, further studies and efforts are needed on various methods of decellularization and their application in vivo. In summary, in tissue engineering application programs, using DBM is still in the process of development. Some post-decellularization processes improve the property of dECM and overcome some deficiencies that occur during bone decellularization. The flexibility of dECM allows it to be processed in a variety of applications, from a complete tissue scaffold to a soluble dECM that can be applied as a bioink for 3D printing. As decellularization technique is a relatively new laboratory process, related devices and technologies are only available on a small laboratory scale. Some research teams focus on standardizing and simplifying decellularization methods, while ameliorating more automated systems with the aim of scaling up the technique and producing a higher volumes of dECM.

## Data Availability

Not applicable.

## Abbreviations

TE: Tissue engineering
dECM: Decellularized exteacellular matrix
ECM: Extracellular matrix
MSCs: Mesenchymal stem cells
BM-MSCs: Bone marrow-derived mesenchymal stem cells
AD-MSCs: Adipose-derived mesenchymal stem cells
BTE: Bone tissue engineering
3D: Three dimensional
FDA: Food and Drug Administration
bECM: Bone Extracellular matrix
OCN: Osteocalcin
OPN: Osteopontin
ON: Osteonectin
FGF23: Fibroblast growth factor 23
BMPs / TGFβ: Bone morphogenetic protein growth factors / beta growth factor superfamily
FGFs: Fibroblast growth factors
VEGFs: Vascular endothelial growth factor
Runx2: Runt-related transcription factor 2
Dlx5: Distal-less homeobox 5
Osx: Osterix
BMPs: Bone morphogenetic proteins
HSCs: Hematopoietic stem cells
BLCs: Bone lining cells
AD-MSCs: Adipose derive mesenchymal stem cells
hDPMSCs: Human dental pulp mesenchymal stem cell
μm: Micrometer
GAGs: Glycosaminoglycans
RGD: Arg-Gly-Asp
DBM: Decellularized bone matrix
SC-CO_2_: Supercritical carbon dioxide
MPa: Megapascal
HHP: High hydrostatic pressure
SDS: Sodium dodecyl sulfate
DNA: Deoxyribonucleic acid
PAA: Peracetic acid
EDTA: Ethylene diamine tetraacetic acid
EGTA: Ethylene glycol tetraacetic acid
DNase: Deoxyribonuclease
RNase: Ribonuclease
dsDNA: Double stranded DNA
SEM/TEM: Scanning/Transfer electron microscopy
HA: Hydroxyl apatite
β-TCP: β-tricalcium phosphate
PCL: Polycaprolactone
NME: Native microenvironment
α-Gal: Galactose-alpha-(1,3)-galactosyl-beta, 4-N acetylglucosamine
BMSC: Bone Marrow Mesenchymal Stem Cells
